# Two-photon emission from a superlattice-based superconducting light-emitting structure

**DOI:** 10.1038/s41377-024-01472-8

**Published:** 2024-06-07

**Authors:** Shlomi Bouscher, Dmitry Panna, Ronen Jacovi, Fauzia Jabeen, Christian Schneider, Sven Höfling, Alex Hayat

**Affiliations:** 1 Department of Electrical Engineering, Technion, Haifa, 32000 Israel; 2 Technische Physik, Universität Würzburg, Am Hubland, D-97074 Würzburg, Germany; 3 Institute of Physics, Carl von Ossietzky Universität Oldenburg, D-26111 Oldenburg, Germany

**Keywords:** Quantum optics, Nonlinear optics

## Abstract

Superconductor-semiconductor hybrid devices can bridge the gap between solid-state-based and photonics-based quantum systems, enabling new hybrid computing schemes, offering increased scalability and robustness. One example for a hybrid device is the superconducting light-emitting diode (SLED). SLEDs have been theoretically shown to emit polarization-entangled photon pairs by utilizing radiative recombination of Cooper pairs. However, the two-photon nature of the emission has not been shown experimentally before. We demonstrate two-photon emission in a GaAs/AlGaAs SLED. Measured electroluminescence spectra reveal unique two-photon superconducting features below the critical temperature (*T*_c_), while temperature-dependent photon-pair correlation experiments (*g*^(2)^(*τ*,*T*)) demonstrate temperature-dependent time coincidences below *T*_c_ between photons emitted from the SLED. Our results pave the way for compact and efficient superconducting quantum light sources and open new directions in light-matter interaction studies.

## Introduction

**Fig. 1:**
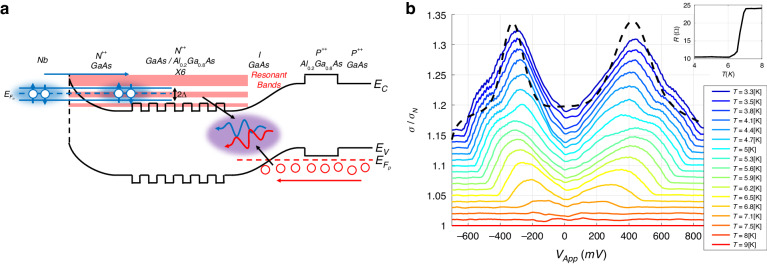
Potential landscape and electrical properties of the superlattice SLED. **a** Schematic drawing of the superlattice SLED; Cooper pairs are injected into the n-type side, recombining with holes injected from the p-type side in both PN junction and n-type layer adjacent to the superconducting contact. The presence of minibands due to the superlattice increases Cooper-pair injection efficiency. **b** Differential conductance measurements σ normalized to a measurement σ_N_ above T_c_, demonstrating Andreev reflection with a calculated dependence (black dashed curve) for the lowest temperature. In order to remove the overlap of the normalized curves and better visualize the features, each curve was shifted vertically by 0.01 relative to the previous curve to improve the clarity of the features. The inset shows the 2-probe measurement of the superconducting contact, demonstrating a superconducting transition around ~6.5 K

Quantum computing and communications schemes are realized by various physical systems including photon-based^1–6^ and solid-state-based platforms^7–14^. While photon-based platforms enable long-distance realizations with minimal losses and dephasing, including various quantum computing^1,5^, teleportation^15,16^, cryptology^17,18^ and metrology^19,20^, extremely weak photon-photon interactions make implementation of various nonlinear photonic quantum gates difficult. On the other hand, while solid-state-based systems often offer a strongly-interacting environment, which enables implementation of quantum gates, dephasing and losses in such systems remain a constant challenge. Hybrid photonic-solid-state systems^21–23^ combine the advantages of both fields, with the photonic aspect enabling long-distance capabilities, while the solid-state aspect provides the required interaction necessary for implementing quantum computing. The superconducting light-emitting diode (SLED)^24,25^ is a hybrid system, which relies on the superconducting condensate coupled to a semiconductor PN junction on the N-type side. When forward bias voltage is applied to the structure, Cooper pairs are injected into the PN junction, where they undergo radiative recombination with holes injected from the P-type side (Fig. 1a), resulting in enhanced emission below the critical temperature *T*_c_ relative to the emission above *T*_c_^25–27^. It was shown theoretically^28^ that a spin-singlet Cooper pair may recombine with a pair of holes, resulting in a polarization-entangled photon pair in the |${\Psi }_{+}$〉 Bell state. This concept has been shown to be also at the core of Cooper-pair based two-photon amplification in waveguides^29^, Bell-state analyzers^30^ and nonlinear photonic universal quantum gates^31^. While the nearly deterministic on-demand sources such as single atoms or quantum dots (QD) offer certain advantages^32–34^, the most widely used source of entangled photons in quantum information processing (QIP) is parametric down conversion (PDC)^5,35–43^, which has a probabilistic nature – very similar to the SLED source. Both PDC and SLED are not based on localized emitters, in contrast to single atoms and QDs, making them non-on-demand sources lacking some of the controllability that QD sources offer. However, this difference allows considerably higher emission rates in both PDC and SLED, with the major advantage of the SLED over PDC being that the SLED is a compact electrically driven device similar to LEDs.

Here, we demonstrate a GaAs-based superlattice PN SLED designed (Fig. 1a) to maximize Cooper-pair injection into the PN junction, via resonant energy bands in the superlattice. We demonstrate enhanced conductance below *T*_c_, a signature indicating Cooper-pair injection, shown by Andreev reflection, into the PN junction. Electroluminescence measurements reveal enhanced emission below *T*_c_, indicating existence of the superconducting order parameter inside the emitting semiconductor layers, as predicted by theory^28,44^. Enhanced emission is predicted to result from two-photon emission, with unique spectral behavior, containing correlated photons pairs. We demonstrate two-photon emission in the electroluminescence spectra, by spectral broadening and shift of the emission spectra below *T*_c_, and photon pair correlations (*g*^(2)^(*τ*,*T*)) below *T*_c_, evidenced by a temperature-dependent peak around time zero, matching our theoretically-modeled dependence.

## Results

Injection of Cooper pairs into the PN junction in SLEDs is evident through enhanced conductance around zero-bias voltage^45^. Andreev reflection is described as an inverse process in which an electron enters the superconducting energy gap, forming a Cooper-pair with another electron, resulting in a hole reflected back. The two-particle nature of the Andreev reflection process makes it highly susceptible to variations in the potential landscape (Schottky barrier) and different materials (Fermi velocity mismatch)^46^, as for both particles, the individual transmission coefficients can be reduced. Both the potential landscape and Fermi velocity mismatch often result in inhibition of Andreev reflection in favor of the quasiparticle tunneling regime, manifesting as reduced conductance inside the superconducting gap rather than enhancement. In order to increase Cooper-pair injection efficiency, bandgap engineering was used and a proper potential landscape was designed^46,47^ to support resonant energy levels close to the superconducting interface. When the quasi Fermi energy level is aligned with one of the resonant energy levels, Cooper-pair injection probability is expected to increase. The superlattice PN structure design is based on a theoretical approach developed in our previous works^46,47^ and is composed of multiple quantum wells (superlattice), resulting in wider resonant energy levels, or minibands (Fig. 1a). The increased width of the minibands eases Fermi energy level alignment. Transport measurements have revealed enhanced conduction below *T*_c_ (Andreev reflection), indicating Cooper-pair injection into the PN junction (Fig. 1b). The conductance curves σ(V) were normalized to a measurement *σ*_N_(V) above *T*_c_ and vertically shifted for clarity, allowing easier observation of the features^48^. The width of the observed enhancement is ~1.4 V, much larger than the expected width of 2Δ, which is on the order of ~mV. The considerable difference is attributed to large nonlinear voltage scaling due to the presence of the nonlinear PN junction in series to the superconductor-semiconductor interface, with the PN junction having much larger resistance, and as a consequence, a much larger voltage drop. The consequent small voltage drop on the superconductor-semiconductor interface results in Cooper-pair injection occurring at higher overall voltages and currents. The shape of the enhanced region is also unique, featuring two large side peaks. These effects are the result of the design of the superlattice. The side peaks result from two peaks located inside one of the mini-bands, with the Fermi energy level located in between them. A theoretical modeling of the transport curve at the lowest temperature is given, which includes the effects of the resonant mini-bands of the superlattice but takes the nonlinear voltage rescaling as constant in temperature (see supplementary for more information).

In contrast to isolated emitters such as QDs emitting photons in a cascade of first-order perturbation processes in specific spectral lines^32,33^, nonlinear optics based sources generate photon pairs by higher-order perturbation processes such as 3rd order in χ^(2)^ based PDC^38,41–43^, 4th order in χ^(3)^ based PDC^49,50^ and 2nd order perturbation in the nonlinear-optical process of two-photon emission in normal materials^51,52^, as well as in the current work on SLEDs. In such nonlinear optical photon pair sources, the emission occurs over a very broad continuous spectral range from zero to the transition energy, with spectral shape and features of this continuum being the most important evidence of the two-photon nature of the emission^39,40,51,53–55^.

**Fig. 2:**
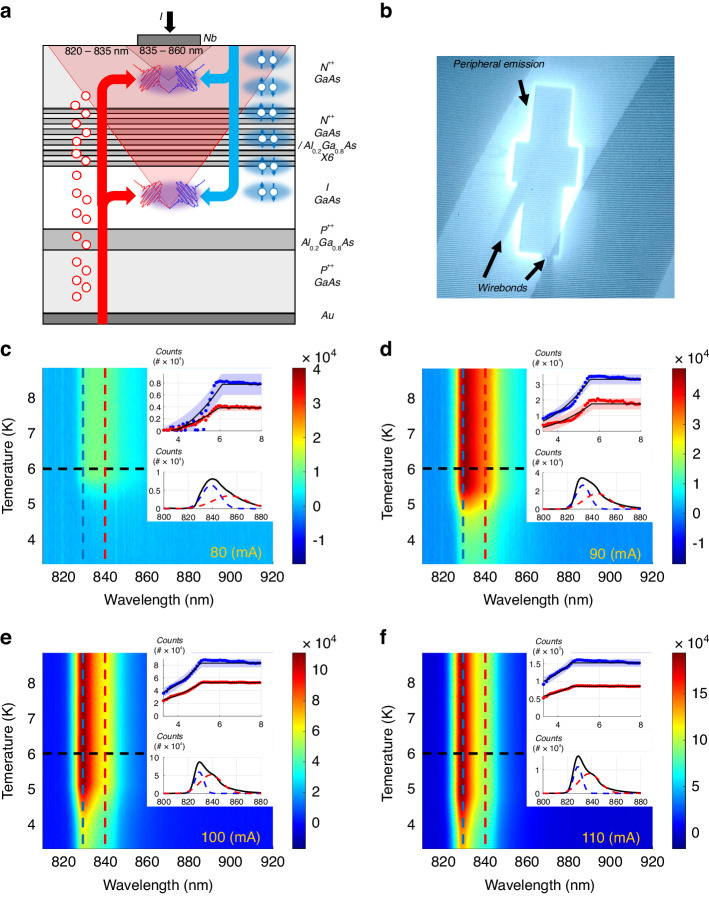
Superlattice SLED emitting regions and EL spectra. **a** Schematic drawing of the emitting regions in the superlattice SLED. Longer wavelength emission arises from the n-type GaAs layer adjacent to the superconducting contact while shorter wavelength emission arises from the intrinsic PN junction. The superconducting contact obscures emission from the top layer due to its thickness. **b** Image of an emitting device. Emission is observed only from the periphery of the device due to the opaqueness of the contact. The diagonal lines are due to the screen’s refresh rate. **c**–**f** Spectral emission of the device vs. temperature for selected current values. All spectra demonstrate decay below T_c_. The vertical dashed blue and red lines mark the positions of the two emission features. The top insets in **c**–**f** depict the decay of the two emission features (blue – shorter wavelength, red – longer wavelength, corresponding to the vertical dashed lines) with temperature and a fit to the expected dependence of Δ^2^(T). The transparent ribbons represent a confidence margin of ±σ for the theoretical fits. The bottom insets in **c**–**f** depict a single recorded spectrum at 6K (black horizontal dashed line), showing the shape of the two features, including the fitting for each feature (blue for shorter wavelength and red for longer wavelength)

In order to observe superconductor-based spectral enhancement, electroluminescence (EL) spectra were measured. The predicted superconductor enhanced two-photon emission was shown theoretically^44^ to depend on the superconducting order parameter Δ squared. As Δ is influenced by both temperature (up to the critical temperature *T*_c_) and current (up to the critical current I_c_), EL measurements were performed for varying currents and temperatures (Fig. 2) to demonstrate the dependence on Δ. A spectrum consisting of two features was observed. The first feature was observed at ~830 nm and is attributed to emission from the intrinsic GaAs layer at the PN junction, while the second feature was observed at ~840 nm and is attributed to emission from the N-type GaAs layer adjacent to the superconductor. The existence of the long-wavelength feature is associated with bandgap shrinkage in the N-type GaAs layer due to the heavy doping^56,57^. A remarkable observation is that below *T*_c_, the emission decreases with decreasing temperature, rather than increase (Fig. 2c–f). We attribute the overall reduction in emission to the superconducting effect on location of emission. Below *T*_c_, emission closer to the contact becomes dominant due to the presence of the superconducting order parameter. Because emission right below the contact is obscured by the contact, the result is a reduction in emission in a trend matching the dependence of the superconducting order parameter with temperature.

**Fig. 3:**
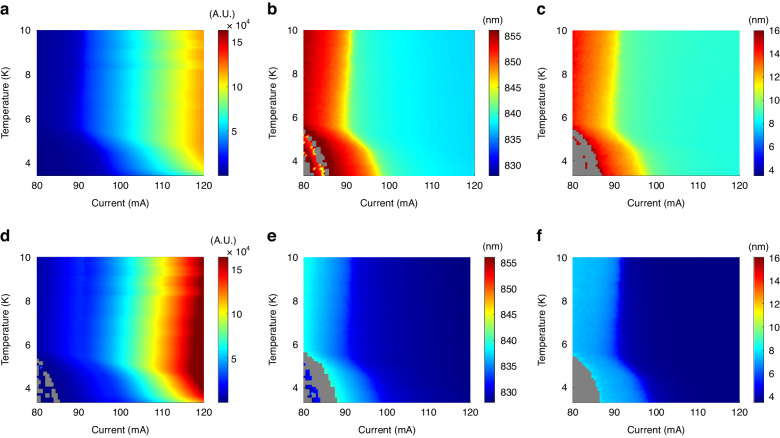
EL spectra feature parameters vs. current and temperature. Spectral feature maximum height (**a**, **d**), center wavelength position (**b**, **e**) and width (**c**, **f**). The top graphs (**a**–**c**) depict the maximum height (**a**), feature position (**b**) and feature width (**c**) of the long-wavelength feature as a function of temperature and current. The bottom graphs (**d**–**f**) depict the maximum height (**d**), feature position (**e**) and feature width (**f**) of the short-wavelength feature as a function of temperature and current. The gray areas represent regions in which no data could be extracted, that is the emission is nonexistent (low current and because emission is closer to the contact)

Superconducting-based changes to the EL spectrum were theoretically predicted by an earlier work^44^ utilizing perturbation theory to first and second order. The first order term describes the contribution of single quasiparticle excitations out of the superconducting state to the EL spectrum. This single particle contribution merely change the EL spectral distribution around the superconducting gap and does not contribute to the enhancement. The second order term describes the two-photon emission process, and is proportional to Δ^2^. The unique features in the emission spectra of our device demonstrate two-photon emission – similar to the widely accepted approach in nonlinear optics^53,54^, and evident in both emission features alongside one-photon emission. We show two-photon emission signature in the EL spectra (Fig. 3), most notably a broadening of the emission below *T*_c_ and *I*_c_(*T*), whose width is proportional to the superconducting order parameter Δ(*T*). The broadening is attributed to the two-photon energies *E*_*1*_,*E*_*2*_, which must fulfill the requirement *E*_*1*_ + *E*_*2*_ *=* *E*_*tot*_, with *E*_*tot*_~2E_gap_, resulting in the observed spectral continuum behavior. Additionally, a shift of the emission towards longer wavelengths was observed. The origin of this shift is attributed to the two-photon emission, as shorter wavelength photons are reabsorbed in the semiconductor layer, causing the weight of the emission to shift towards longer wavelengths. The shift, like the broadening, is proportional to the superconducting order parameter Δ(*T*). Furthermore, changes in emission strength below *T*_c_ and *I*_c_(*T*) are also observed. The changes in emission are proportional to the superconducting order parameter temperature dependence.

From Fig. 3, it is observed that for both spectral features, a shift towards longer wavelengths is observed, as well as an increase in the width. Both parameters show a superconducting-dome-like behavior. The increased width of both spectral features below *T*_c_ and *I*_c_(*T*) is attributed to the appearance of the broadened two-photon emission spectrum^28^. In addition, for the two-photon continuum spectra, photons with energies higher than E_gap_ may be emitted. However, because of their large energy, they are expected to be absorbed in the GaAs layers, shifting the emission to longer wavelengths. Both effects are observed to be proportional to the superconducting order parameter Δ(*T*), vanishing above *T*_c_, in agreement with theory.

**Fig. 4:**
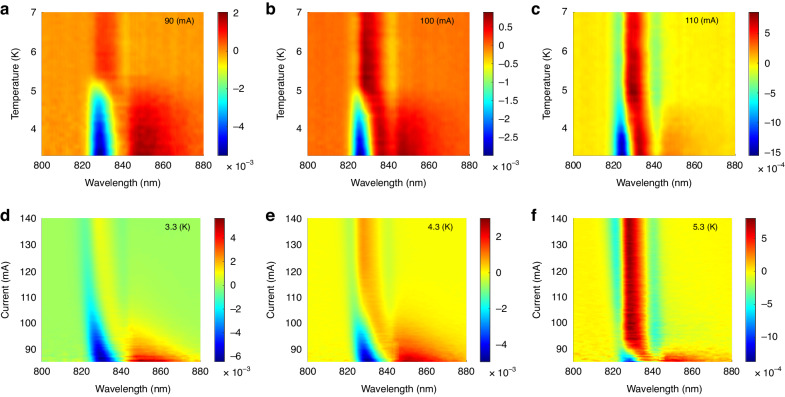
Normalized EL spectra vs. current and temperature. **a**–**c** Normalized spectra vs. temperature for different currents. **d**–**f** Normalized spectra vs. current for different temperatures. For both types of spectra, a peak is observed at longer wavelengths (840–860 nm) while a dip is observed at shorter wavelengths (820–840 nm)

As the emission spectrum is composed of two features, whose emission is attributed to two distinct layers, the ratio between both features is expected to change with varying temperature or current. In order to demonstrate the interplay between the features, each EL spectral curve was normalized by the spectrum at 10 K, above *T*_c_ (Fig. 4). Because the superconducting proximity effect is expected to increase in magnitude closer to the superconducting contact, enhancement is expected to occur in the adjacent degenerate GaAs layer, favoring emission at longer wavelengths over shorter wavelengths below *T*_c_ and *I*_c_. Enhancement at longer wavelengths was observed below *T*_c_ and *I*_c_, together with reduced emission at shorter wavelengths, due to the stronger presence of the superconducting order parameter close to the superconducting contact, and the corresponding absorption. The two dominant features in Fig. 4 are uneven, with the peak feature being weaker than the dip feature. The difference in the uneven distribution is due to the difference in depth of emission of either feature. We attribute the long wavelength feature (840–860 nm) to emission closer to the contact and the short wavelength feature (820–840 nm) to emission farther than the contact. As emission closer to the contact is more obscured, the long wavelength feature is expected to be weaker in comparison to the short wavelength feature, explaining the uneven distribution between the two.

**Fig. 5:**
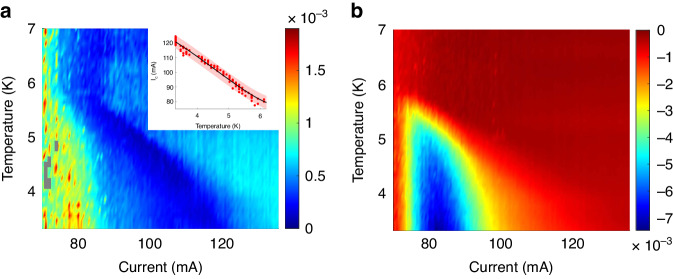
Normalized EL spectra maximum and minimum vs. current and temperature. **a** Value of the peak of the normalized spectra vs. temperature and applied current, having a superconducting-dome-like signature. The inset depicts the critical current-temperature relation, the solid black line is the calculated dependence, with the transparent red ribbon representing a confidence margin of ±σ. **b** Value of the dip of the normalized spectra vs. temperature and applied current, having a superconducting-dome-like signature

For the normalized spectral curves, the values of the peak and dip were extracted (Fig. 5). The existence of the peak indicates enhancement, corresponding to superconductor-enhanced emission closer to the superconducting contact, as predicted by theory^44^. The dip is attributed to the reduction of emission further than the superconducting contact. Because of the enhanced emission dependence on the superconducting gap, both dip and peak values are expected to share similar trends with respect to current and temperature, forming a superconducting-dome-like dependence.

Extracted normalized maximum and minimum EL values demonstrate a superconducting-dome-like shape with respect to current and temperature, vanishing above the critical current and temperature of the superconductor. The dependence of *I*_c_ vs. sample temperature was extracted for the peak (Fig. 5a inset), closely matching theoretical prediction^58^. The superconducting-dome-like shape of the peak further demonstrates that the enhanced emission is proportional to Δ^2^, and is superconducting in origin.

In our experiments, two-photon emission was observed as a broadening of the emission spectrum below *T*_c_ and *I*_c_(*T*). The observed broadening was proportional to the superconducting order parameter Δ(*T*), demonstrating both the two-photon nature of the emission as well as its superconducting origin through radiative Cooper-pair/hole-pair recombination. This observation matches theoretical predictions^28^. In addition, the EL spectra demonstrated a shift towards longer wavelengths below *T*_c_ and *I*_c_(*T*), due to higher energy photons becoming reabsorbed in the semiconductor stack, as expected to occur for a broadened two-photon emission, and was also observed to be proportional to the superconducting order parameter Δ(*T*). Moreover, the EL spectral shape demonstrates clear dependence on both *T*_c_ and *I*_c_(*T*), becoming independent of temperature above either *T*_c_ or for currents above *I*_c_(*T*). The decrease in the magnitude of the EL spectrum below the critical temperature *T*_c_ follows a trend that matches the superconducting order parameter Δ(*T*) dependence on temperature. Finally, the interplay between the two emission features of the EL spectrum, with the dominance of the long wavelength feature at low currents and temperatures, demonstrates superconducting-originating emission enhancement, which was previously demonstrated^24,59^. The two-photon emission presented in our device is stable to within 1.5%, during many days of measurements, and exhibits repeatable results under temperature and current cycles, however in order to avoid damage to the device due to excess current and joule heating, the device is operated in a pulsed regime in order to ensure low and stable operating temperature. We attribute the stability margin of our device to the stability of the current source used in the measurement.

Furthermore, we characterized the statistical properties of the two-photon emission by performing correlation (*g*^(2)^(*τ*,*T*)) measurements using a Hanbury Brown–Twiss setup^60^, with τ being the difference in photon detection times. The expression for the correlation *g*^(2)^(*τ*,*T*) function is^61^

1 $${g}^{(2)}(\tau ,T)=\frac{\left\langle {{a}}_{1}^{\dagger }(t){{a}}_{2}^{\dagger }(t+\tau ){{a}}_{2}(t+\tau ){{a}}_{1}(t)\right\rangle }{\left\langle {{a}}_{1}^{\dagger }(t){{a}}_{1}(t)\right\rangle \left\langle {{a}}_{2}^{\dagger }(t+\tau ){{a}}_{2}(t+\tau )\right\rangle }$$

Where ${\hat{a}}_{i}^{\dagger }(t),{\hat{a}}_{i}(t)$ are photon creation and annihilation operators, and 〈 〉 is a time and ensemble average. Two-photon correlation visibility depends on the emission rate, with high rates resulting in strong one-photon accidental photon coincidence background, necessitating operation at lower rates. As a result, a structure that can optimize two-photon emission over one-photon emission is required. The superlattice structure is intended to maximize the Cooper-pair injection rate at low voltages, increasing the ratio of two-photon to one-photon emission. This occurs by the superlattice structure forming resonant energy minibands with a high transmission coefficient. The high transmission coefficient results in improved Cooper-pair injection, as was demonstrated in an earlier work^46^. Nevertheless, two-photon emission from superlattice superconducting structures contains two key challenges. First, two-photon emission is accompanied by one-photon emission, reducing the visibility of photon coincidences. Both types of emission exhibit a broad spectrum, making filtering of suitable wavelengths a challenging task. Second, a considerable portion of the emission is obscured by the superconducting contact. In addition, the difference between the effective indices of the GaAs/AlGaAs superlattice stack and vacuum further limit the extraction efficiency of the device.

**Fig. 6:**
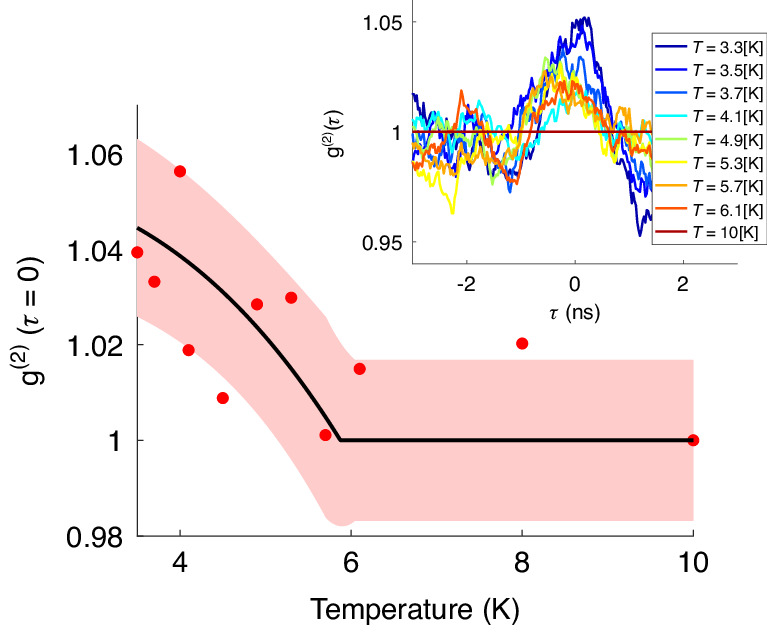
Photon coincidence measurements of superlattice SLED emission. g^(2)^(τ = 0,T), indicating photon-pair correlations below T_c_. The black curve is a calculated dependence. The transparent red ribbon represents a confidence margin of ±σ for the theoretical model. The inset shows g^(2)^(τ) vs. temperature. A peak is observed at τ = 0 with a width of ~0.5 ns, matching the total jitter of both APDs

To clearly demonstrate photon correlations, uncorrelated one-photon emission must be kept to a minimum. In the SLED, one-photon emission increases considerably at high currents due to a much lower injection ratio of Cooper pairs over single electrons, implying current must be kept low. Because the voltage drop on the PN junction is much larger than the voltage drop on the superconductor-semiconductor junction (Fig. 1b), the gradient of the electron quasi-Fermi energy level inside the superlattice is expected to be small. As a result, larger voltages can be applied with the quasi-Fermi energy level still being located inside the superlattice miniband, maintaining high Cooper-pair injection efficiency due to resonant tunneling, and keeping single electron injection low. Cooper pair to single electron injection ratio can thus be kept sufficiently high at higher currents such that the current may be increased for the benefit of stronger emission. Because increased current implies more Joule heating, the current is pulsed, with pulse width being sufficiently large (»100 µs) to prevent distortion of *g*^(2)^(*τ*). The EL spectra also provide information on the current-temperature work point where the strongest correlation signal is expected, as can be seen from the resulting superconducting dome-like shape of the two-photon peak (Figs. 3 and 5). In the process of two-photon emission, the coherence time is the inverse of the bandwidth of the emitted photons. Most of the emission is concentrated within a ~10–20 meV span (Figs. 2 and 3), yielding a coherence time of ~250 fs. However, the avalanche photo diode (APDs) timing jitter is ~0.35 ns per APD, resulting in a broadening of the *g*^(2)^(*τ*) peak to a width of ~0.5 ns (due to the overall timing jitter of both APDs). Below *T*_c_, a temperature-dependent peak at *g*^(2)^(*τ* = 0) was observed (Fig. 6), indicating existence of photon-pair correlations. The peak reached a maximum value of 1.06, having a width of ~0.5 ns, matching APD timing resolution.

The *g*^(2)^(*τ* = 0,*T*) curve follows a trend proportional to Δ^2^(*T*), matching the theoretical prediction^28^. The extracted critical temperature is ~6 K, similar to values obtained from the transport and EL data. The contribution of one-photon emission to *g*^(2)^(*τ*,*T*) is expected to scale like *n*^2^, where n is the rate of emitted photons. The two-photon emission contribution to *g*^(2)^(*τ*,*T*) is expected to scale like *n*. For high photon emission rates, because the one-photon contribution scales faster with the rate of emitted photons, it is expected to create a strong background, reducing the size of the correlations originating from the two-photon component, thus explaining the small value of the peak. Above *T*_c_ or *I*_c_(*T*), the pair-generation rate vanishes. In addition, the ratio between the one-photon-generation rate and two-photon generation rate depends on the current, as the current changes the potential landscape of the device, resulting in varying levels of Cooper-pair injection into the device and subsequent generation rates for both one and two-photon emission.

## Discussion

While enhanced emission below *T*_c_ was previously observed in superconductor-coupled PN junctions^24,25,27,59^ and superconductor-coupled quantum dots^26^, the origin of the observed enhancement was attributed to modification of the host material’s density of states by the parent superconductor as well as recombination of Cooper pairs^26^. In our work, we observe, for the first time, clear features which can be directly attributed to the two-photon nature of the emission. Two-photon emission can be observed through four unique signatures: First, we observe broadening of the EL spectrum below *T*_c_ and *I*_c_(*T*). The origin of the broadening is attributed to both the continuum of the photon energies in a second-order process and the Cooper-pair related energy structure of the superconductor. Second, the EL spectra shifts towards longer wavelengths below *T*_c_ and *I*_c_(*T*), due to higher energy photons becoming reabsorbed in the semiconductor stack, as expected to occur for a broadened two-photon emission. Third, two-photon emission results in photon-pair correlations *g*^(2)^(*τ*,*T*) below *T*_c_ and *I*_c_(*T*). So far, Cooper-pair injection efficiencies in superconductor-semiconductor structure were limited due to the potential barrier at the superconductor-semiconductor interface. Using our potential landscape engineering method resulting in resonant-tunneling structures, we have successfully obtained high Cooper-pair injection efficiencies^46,47^, allowing us to reach the proper Cooper-pair injection regime enabling observation of two-photon emission. We have observed all of the above signatures in our superconductor superlattice device.

In conclusion, we demonstrated Cooper-pair two-photon emission. Cooper pair injection, evident as electric conductance enhancement below *T*_c_. Unique two-photon spectral features, such as spectral broadening and spectral shift, were also observed below *T*_c_, with enhanced emission obtained at longer wavelengths, for bias currents and temperatures smaller than *I*_c_ and *T*_c_. The dependence of the emission on both current and temperature revealed a superconducting-dome-like structure, indicating the superconducting origin of the enhanced spectrum. Finally, *g*^(2)^(*τ*,*T*) measurements have revealed photon coincidences, indicating preference for pair emission in our devices. The demonstration of two-photon emission in SLED devices paves the way for a new generation of hybrid superconductor-semiconductor devices with many applications in the fields of quantum computing and quantum information processing.

## Materials and methods

### Sample design, fabrication and packaging

The semiconducting stack was designed with the aim of maximizing Cooper-pair injection^45,46^. The semiconductor stack was grown using molecular beam epitaxy (MBE) on a GaAs substrate. A 200 nm Nb layer was then deposited via sputtering on the sample, and subsequent pads were fabricated using standard photolithography and reactive ion etching (RIE). The samples were bonded to a LCC28 chip holder using a wire-bonder and then inserted into a suitable cryogenic environment.

### Electrical measurements

Electrical transport measurements were performed using a lock-in amplifier in a 4-probe configuration. The AC frequency was selected to reduce measure measurement noise to a minimum. DC work currents were kept low (<15 mA) in order to avoid Joule heating.

### Electroluminescence and *g*^(2)^(*τ*,*T*) experiment setup

Both electroluminescence and *g*^(2)^(*τ*,*T*) measurements were performed using a current source, in pulsed mode, in order to maximize the bias-current while keeping device heating to a minimum, allowing for an independent measurement of the spectral properties of the samples with changing bias current and temperature. For the correlation measurement, special care was taken to ensure a low duty-cycle but a long enough current pulse (>100 µs) in order to avoid distortion of the *g*^(2)^(*τ*,*T*) measurement due to the current pulses. A 50–50 beam-splitter, two APDs (total jitter of ~0.5 ns) and a time-tagger unit, were used for the *g*^(2)^(*τ*,*T*) measurement. The entire setup was sealed inside a black box in order to reduce dark counts to minimum. Because the APDs emit photons after each detection event, parasitic correlations are induced. Therefore, the optical path was increased so that the unwanted correlation peaks will be located much further than *τ* = 0 (~10–20 ns).

### Electroluminescence experiment fitting

As a double-feature structure was observed in the EL spectrum S(λ) (Fig. 2c–f), it was fitted using a double Gaussian

2 $$S(\lambda )=a\cdot {e}^{-\frac{{(x-b)}^{2}}{2{c}^{2}}}+d\cdot {e}^{-\frac{{(x-e)}^{2}}{2{f}^{2}}}$$

where $a,b,c,d,e,f$ are fitting coefficients. The strengths of both emission features ($a,d$) were then obtained, allowing the extraction of their decay below *T*_c_. The relation between the critical current I_c_ and temperature was modeled after Bardeen’s work^58^

3 $${I}_{c}(T)={I}_{{c}_{0}}\cdot {\left[1-{\left(\frac{T}{{T}_{c}}\right)}^{2}\right]}^{3/2}$$

Where ${I}_{{c}_{0}}$ and *T*_c_ are fitting parameters accounting for the critical current at 0 K and the critical temperature respectively.

### Supplementary information

Supplementary Material of Two-photon emission from a superlattice-based superconducting light-emitting structure

## Data Availability

All data needed to evaluate the conclusions in the paper are present in the paper and/or the Supplementary Materials.
