# Supplementary material for: Two-photon emission from a superlattice-based superconducting light-emitting structure
Source: Light Sci Appl. 2024 Jun 7;13:135. doi: 10.1038/s41377-024-01472-8 (PMC11161636; doi:10.1038/s41377-024-01472-8)
Supplement: Supplementary file 1 — Supplementary Material of Two-photon emission from a superlattice-based superconducting light-emitting structure [file 41377_2024_1472_MOESM1_ESM.pdf]

Supplementary Information for

Two-photon emission from a superlattice-based superconducting light-emitting structure

Shlomi Bouscher[1], Dmitry Panna[1], Ronen Jacovi[1], Fauzia Jabeen[2], Christian Schneider[2], Sven Höfling[3] and Alex Hayat[1*]

[1]*Department of Electrical Engineering, Technion; Haifa 32000, Israel*
[2]*Technische Physik, Universität Würzburg; Am Hubland, D-97074 Würzburg, Germany*
[3]*Institute of Physics, Carl von Ossietzky Universität Oldenburg; D-26111 Oldenburg, Germany*

**Corresponding author. Email: alex.hayat@ee.technion.ac.il*

Introduction

The purpose of this supplementary document is to provide further details regarding the various aspects of the experiment and theoretical modelling: Potential barrier modelling and spectral feature extraction.

Potential barrier modelling parameters

In order to model the effects of the superlattice structure on the transport spectrum, we have used an extended version of the Blonder-Tinkham-Klapwijk (BTK) model[1], which utilizes the scattering matrix approach to account for both spatially varying potential and superconducting parameter[2]. An example to the utilization of our extended model appears in a previous work[3] and its corresponding supplementary material. The superlattice superconducting light-emitting diode (SLED) is composed of two junctions: the superconductor-semiconductor junction, which includes the interface and superlattice regions, and the PN junction, which includes the intrinsic and p-type regions. The intrinsic and p-type regions form an infinitely-long barrier, which is expected to inhibit transmission altogether. In practice, charge carriers can undergo recombination in the intrinsic region, resulting in non-zero transmission evident as current in our device. As our model does not

include the effects of recombination, the intrinsic and p-type regions have been replaced with a narrow triangular barrier intended to simulate the overall confining nature of the larger quantum well, in which the superlattice structure is found. The expected potential landscape and the simulated potential landscape are given in Figure S1 along with the modelling parameters for the simulated potential landscape.

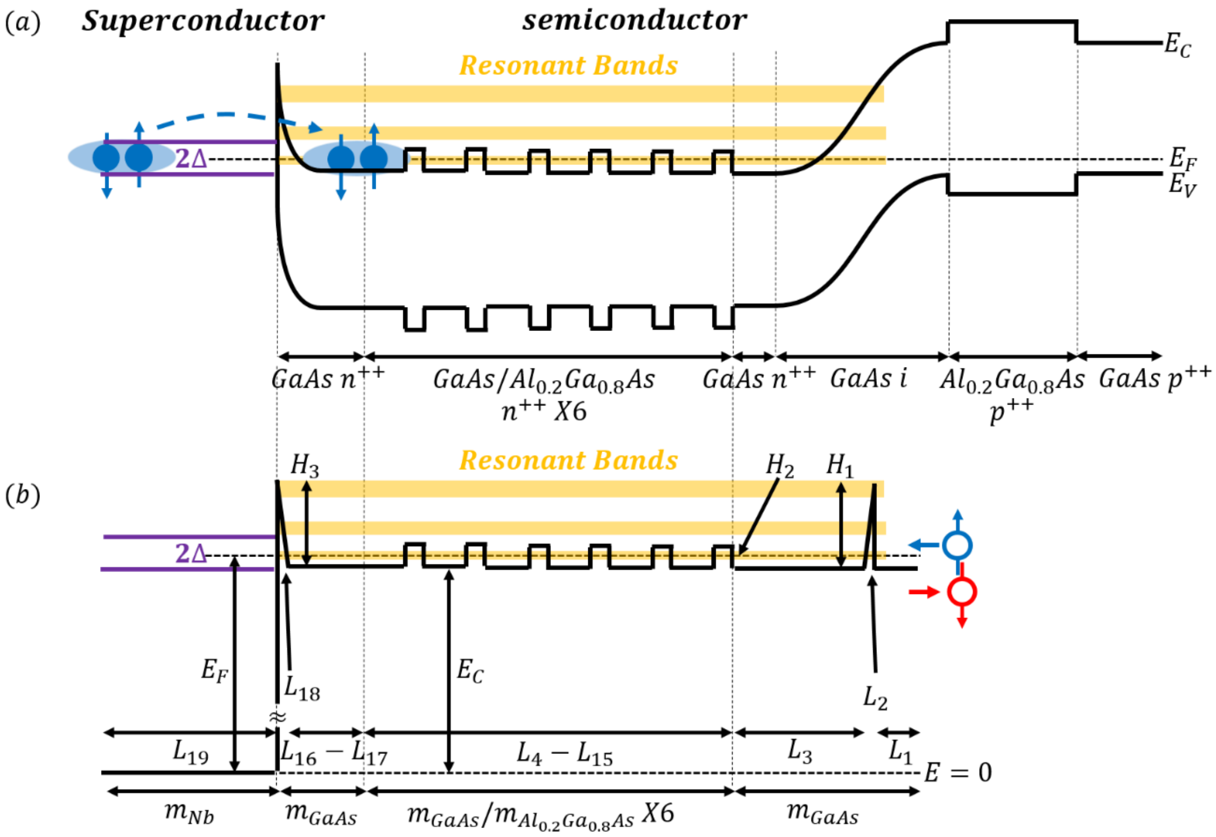

*Figure S1 – **Superlattice SLED potential landscape and modelling:** (a) Energy band description of the superlattice-based SLED (b) Modelling scheme for the device depicted in (a). Note that the PN junction is replaced with a triangular barrier in order to simulate the arrival of charge carriers from the depletion region.*

The various parameters are provided in a table below (Table 1).

Parameter	Value	Description
L_1	$1\ nm$	Pre PN barrier length (replaces p-type/intrinsic layers) – designed
L_2	$1\ nm$	PN barrier thickness (replaces p-type/intrinsic layers) - designed
L_3	$10\ nm$	Pre superlattice thickness - designed
$L_4, L_6, L_8, L_{10}, L_{12}, L_{14}$	$2\ nm$	Superlattice $Al_{0.2}Ga_{0.8}As$ thickness - designed

$L_5, L_7, L_8, L_{11}, L_{13}, L_{15}$	$10\ nm$	Superlattice GaAs thickness - designed
L_{16}	$5\ nm$	Post superlattice thickness 1 - designed
L_{17}	$4.5\ nm$	Post superlattice thickness 2 - designed
L_{18}	$1\ nm$	Schottky barrier thickness (part of interface Schottky barrier) - fitted
L_{19}	$100\ nm$	Nb thickness - designed
H_1	$0.8\ eV$	PN barrier height (replaces p-type/intrinsic layers) - designed
H_2	$0.2\ eV$	Superlattice $Al_{0.2}Ga_{0.8}As$ height - fitted
H_3	$0.9\ eV$	Schottky barrier height - designed
$E_F - E_C$	$90\ meV$	GaAs conduction band edge relative to Nb Fermi energy - fitted
E_F	$5.2\ eV$	Nb Fermi energy level[4]
m_{GaAs}	0.067	GaAs effective electron mass ratio[5]
$m_{Al_{0.2}Ga_{0.8}As}$	0.08	$Al_{0.2}Ga_{0.8}As$ effective electron mass ratio[5]
m_{Nb}	1.8	Nb effective electron mass ratio[6]
Δ	$1\ meV$ @$3.2\ K$	Superconducting order parameter - fitted

Table 1 – Design and fitting parameters for the superlattice device.

The final design specifications of the entire device are detailed in the table below (Table 2).

Layer	Material	Thickness (Angstrom)	Doping Type	Doping Concentration (cm^{-3})	Comments
8	Nb	2000			Superconductor
7	GaAs	50	N^{++}	$5\cdot10^{19}$	High doping for Ohmic contact
6	GaAs	50	N^{++}	$3\cdot10^{18}$	
5	GaAs	100	N^{++}	$3\cdot10^{18}$	Super-lattice – X6
4	$Al_{0.2}Ga_{0.8}As$	20	N^{++}	$3\cdot10^{18}$	Super-lattice – X6
3	GaAs	300	N^{++}	$3\cdot10^{18}$	
2	GaAs	300	I		Intrinsic and Active emitter layer
1	$Al_{0.2}Ga_{0.8}As$	5000	P^{++}	$5\cdot10^{18}$	Hole confinement layer
0	GaAs	---	P^{++}	$5\cdot10^{18}$	P-Type Substrate (001)

Table 2 – Superlattice stack thicknesses.

Using our model and the parameters above, the probabilities $A(E), B(E)$ were calculated. $A(E)$ represents the hole reflection probability (Andreev reflection), while $B(E)$ represents the electron reflection probability. Because of the superlattice structure's inherent complexity, the overall transmission term of the device $1 - B(E) + A(E)$ has been convoluted with a normalized Gaussian distribution with standard deviation $\alpha + \beta(T)$ (Table 3) in order to incorporate effects of disorder and temperature. Both current and electrical conductance of our device can then be approximated by the following equations[1]

$$I_{Semi->Super} = 2N(0)ev_{Fermi}A_{Junction}\int_{-\infty}^{+\infty}\left[f_0(E-eV_{Applied}) - f_0(E)\right][1-B(E)+A(E)]dE$$
$$\frac{\partial I_{Semi->Super}}{\partial V_{Applied}} = 2N(0)ev_{Fermi}A_{Junction}\int_{-\infty}^{+\infty}\left[\frac{df_0(E-eV_{Applied})}{dV_{Applied}}\right][1-B(E)+A(E)]dE \quad (1)$$

Where

$$f_0(E-eV_{Applied}) = \frac{1}{1+e^{\frac{E-eV_{Applied}}{k_BT}}}$$
$$\frac{df_0(E-eV_{Applied})}{dV_{Applied}} = \frac{\frac{e}{k_BT}\mathrm{e}^{\frac{E-eV_{Applied}}{k_BT}}}{(1+\mathrm{e}^{\frac{E-eV_{Applied}}{k_BT}})^2} \quad (2)$$

is the Fermi-Dirac distribution and its derivative relative to the voltage $V_{Applied}$. The parameters $A_{Junction}, v_{Fermi}, e, N(0)$ are the area of the junction ($\sim 50 \times 100\ \mu m^2$), Fermi velocity in Nb, electron charge and single particle density of states of Nb at the Fermi energy level. All of the above coefficients are combined into $\chi = 2N(0)ev_{Fermi}A_{Junction}$ which serves as a fitting parameter with units of $\frac{A}{J}$.

Fitting Parameter	Value	Description
α	$6\,meV$	Constant disorder parameter - fitted
β	k_BT	Temperature-dependent broadening parameter.
χ	$0.7 \cdot 10^{19}\ \frac{A}{J}$	Superconductor-Semiconductor current scaling parameter - fitted

Table 3 – Disorder and current scaling parameters.

As our modelling only simulates the superconductor-semiconductor junction, the contribution of the PN junction to the overall *I*-*V* and σ-*V* curves must be extrapolated from the experimental *I*-*V* and σ-*V* curves above T_c. The PN junction was assumed to behave as a nonlinear resistor in series to the superconductor-semiconductor junction, with the effect of nonlinearly stretching the superconductor-semiconductor *I*-*V* curves. Of note, an additional existing model is the Landauer-Büttiker model[7,8]. It is more general than the BTK model[1,2], which does not take into account scattering between different modes of the potential landscape. We chose to use the BTK formalism because for niobium, the superconducting order parameter is isotropic, implying that transitions between different orientations of plane-wave modes does not carry any additional information about the behavior of the system. This is in contrast to high-T_c superconductors, where the superconducting order parameter does not have to be isotropic, requiring the use of models that include transitions between different plane wave modes[9].

Feature extraction from the electroluminescence spectra

The electroluminescence (EL) spectra exhibit a double feature structure indicating emission from two distinct regions in our device (figure 2a in the main text). In order to extract the position, height and width of each feature, a double Gaussian shape was assumed for the spectrum

$$S(\lambda) = ae^{-\frac{(\lambda-b)^2}{2c^2}} + de^{-\frac{(\lambda-e)^2}{2f^2}} \tag{3}$$

Where a, d represent the height of both feature, b, e represent the position of both feature and c, f represent the width of both feature. An example for a fit along with the tracing of the feature positions vs. temperature is given in Figure S2 below. The extracted feature heights were then plotted vs. temperature to demonstrate the superconducting signature evident in the decaying spectrum.

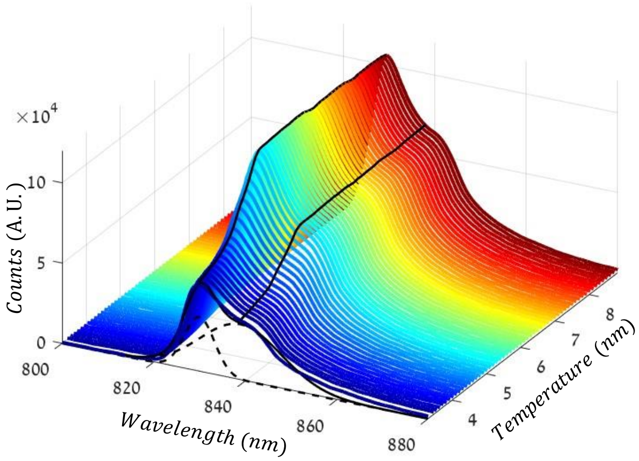

*Figure S2 – **EL Spectra fitting:** Spectra vs. temperature for 100mA pulsed current. The dashed lines mark the fitting of both gaussians for the lowest temperature with the solid black line marking the overall spectral fitting. The two black curves on top of the spectrum are plot the extracted feature positions as a function of temperature.*

For each of the fitting parameters, the best fit as well as the 95% confidence intervals were extracted, and appear in Figure S3 below.

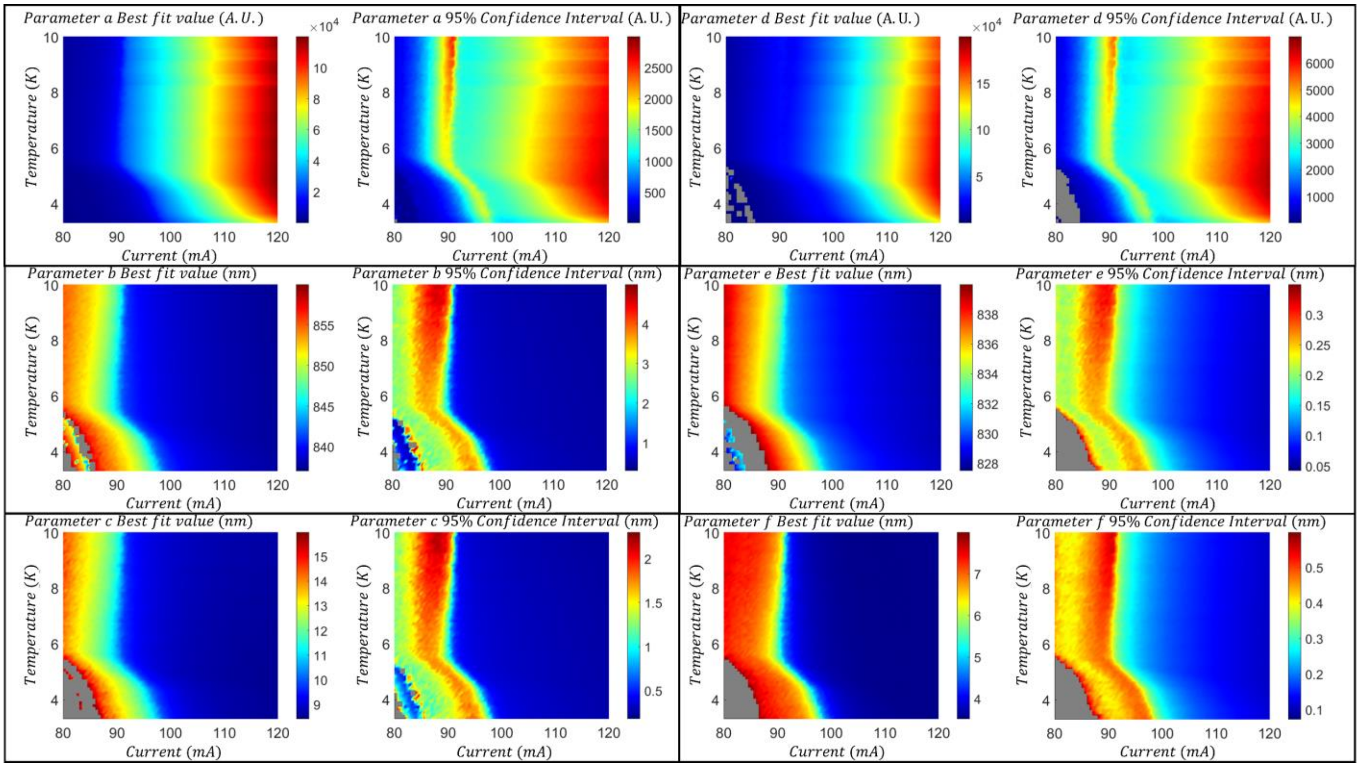

*Figure S3 – **EL spectra fitting parameters and confidence intervals:** Double gaussian fitting parameters vs. current and temperature. For each fitting parameter, the best fitted value and the 95% confidence intervals are plotted.*

Additional electroluminescence data

The figures below include additional EL data not presented in the main text, with the purpose of better highlighting the trends discussed. The data is divided in two: EL measurements taken for constant currents and varying temperatures, and EL measurements taken for constant temperatures and varying currents. For small injection currents (80-105 mA), enhancement at longer wavelengths (850-860 nm) is evident. For large injection currents (110-135 mA) the enhancement reduces in strength until it finally disappears (Figure S4). Moreover, for large injection currents, an-enhancement-like feature is observed between two dips. This feature is not superconducting in origin as it persists well above T_c. Instead, the origin of this feature is due to the Fermi-Dirac distribution and normalization of the spectrum to above T_c. At low temperatures, the Fermi-Dirac distribution becomes sharper. Therefore, normalization of the Fermi-Dirac distribution at low temperature to the Fermi-Dirac distribution at high temperature will induce a peak and a dip structure, as evident in the spectral curves. The same behavior can also be observed for constant temperature and changing currents (Figure S5).

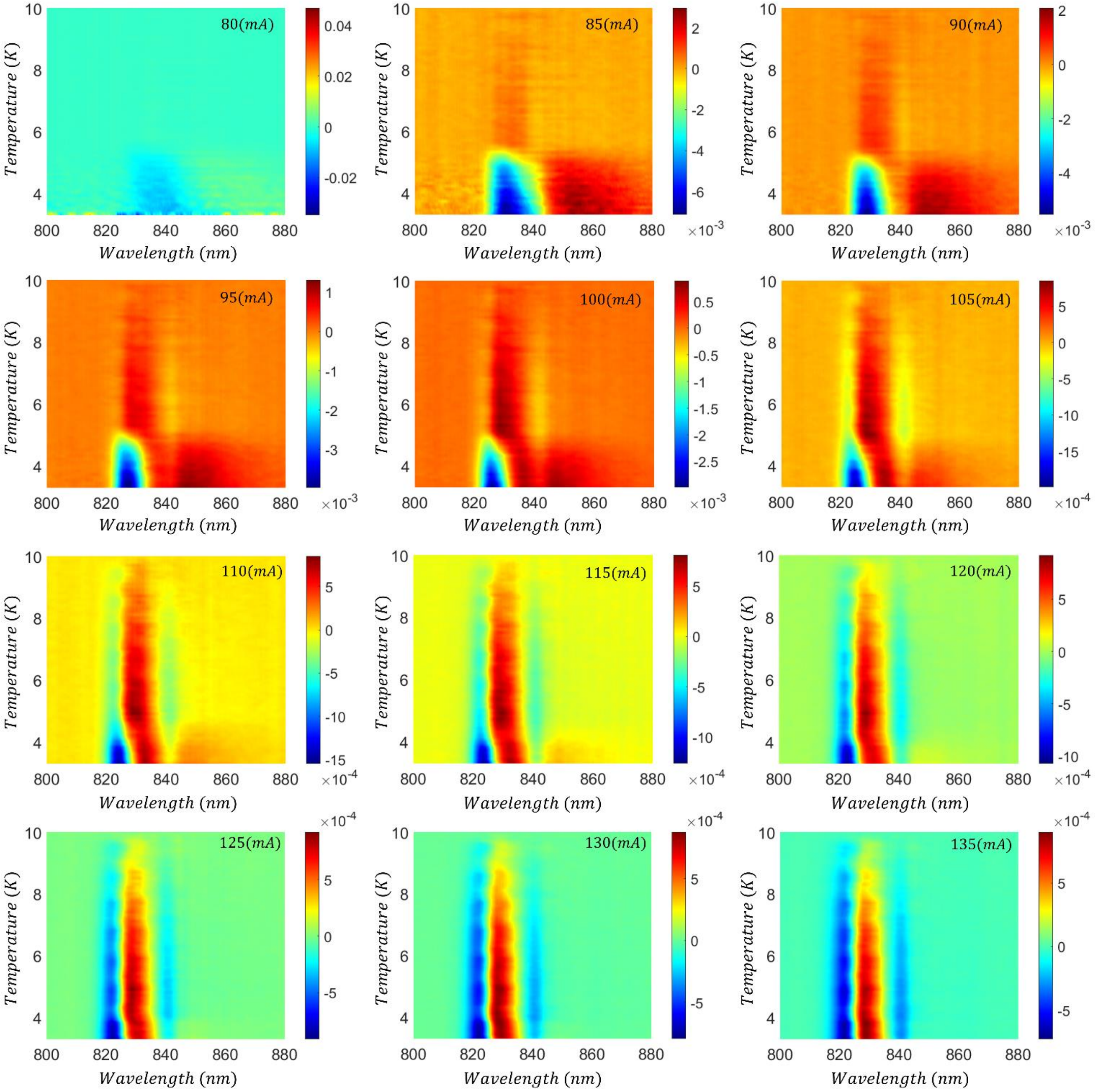

Figure S4 – ***Extended plots of normalized EL spectra vs. temperature:*** *Temperature-dependent electroluminescence spectra at set bias currents for the superlattice PN junction sample. At low bias-currents and temperatures, enhanced emission at longer wavelengths can be observed.*

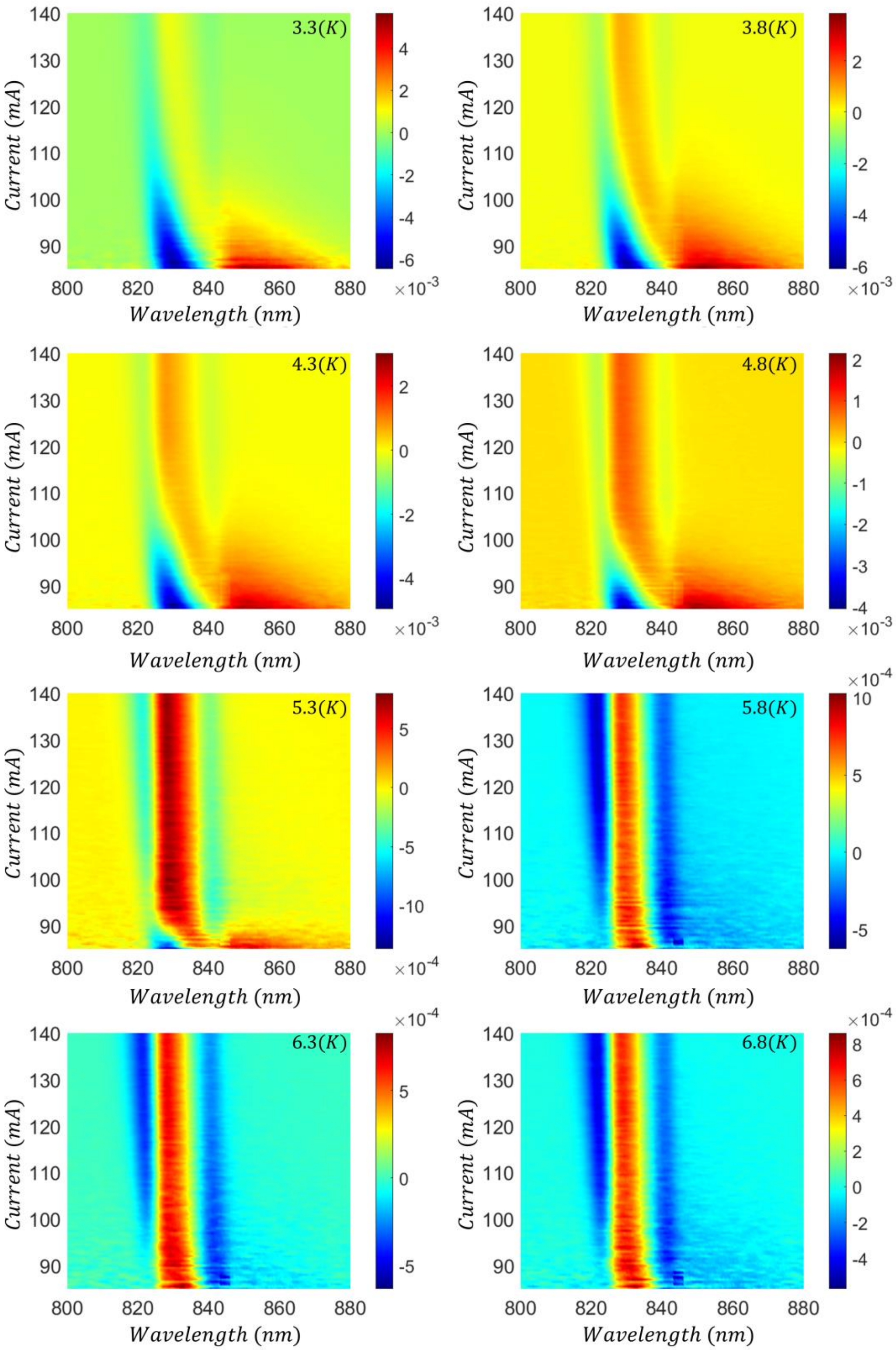

Figure S5 - ***Extended plots of normalized EL spectra vs. current:*** *Current-dependent electroluminescence spectra at set temperatures for the superlattice PN junction sample. At low bias-currents and temperatures, enhanced emission at longer wavelengths can be observed.*

Superconducting order parameter approximation

The equation used to approximate the superconducting order parameter $\Delta(T)$[10,11,12] is

$$\Delta(T) = \begin{cases} \Delta_0 \tanh\left[1.74\sqrt{\frac{T_c}{T}-1}\right] & ,T \le T_c \\ 0 & ,T > T_c \end{cases} \qquad (4)$$

The approximate dependence of $\Delta(T)$ was used in the fitting of the short and long wavelength peaks (figure 2c-f top inset) as well as used in the modelling of the 2nd order coherence peak $g^{(2)}(\tau=0,T)$.

Modelling the device extraction efficiency and intensity correction

A round pad with diameter D_{Pad} is assumed for simplicity. Emission is assumed to occur from a cylindrical region directly below the pad with the same lateral dimensions (cross-section) as the pad. In this case, emission can only extracted from the periphery of the cross-section (Figure S6). This is because the pad blocks any direct vertical emission.

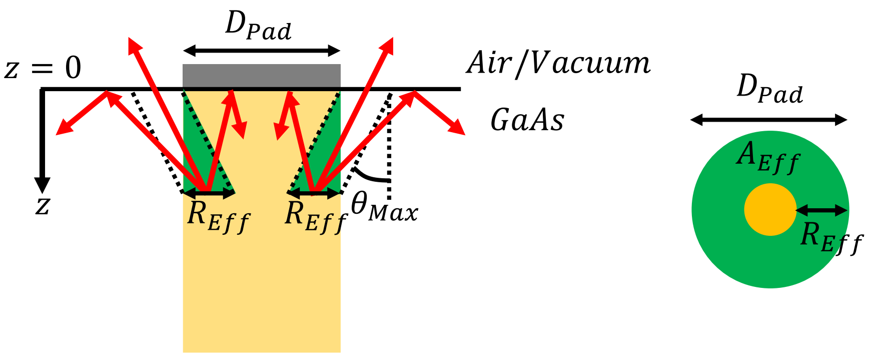

*Figure S6 - **Geometric model for extraction efficiency:** Left: Emission is assumed to occur from a cross-section the size of the contact (orange region). Emission is either blocked by the contact or by the critical angle at the GaAs-Air/Vacuum interface. At a result, the only emission that is extracted is from a region with a width R_{Eff}. Right: top view of the effective emitting region. The pad is assumed to be circular for simplicity of calculations.*

In addition, emission is limited by the critical angle θ_{Max} between the GaAs layer and the air layer. For wavelengths in the range of 800-850 nm, GaAs has a refractive index of ~3.66 (ref. 5). θ_{Max} can be calculated from Snell's law

$$\theta_{Max} = \arcsin\left(\frac{1}{n_{GaAs}}\right) \sim 15.85^{\circ} \qquad (5)$$

Defining z as the depth below the contact from which emission occurs, R_{Eff}, is defined as the fraction of the pad's radius close to the periphery of the pad, from which emission can be extracted. R_{Eff} has the following relation to z and the critical angle

$$R_{Eff}(z) = z \cdot \tan\theta_{Max} \qquad (6)$$

Because in our devices D_{Pad}~100 µm, z~100 nm, we can approximate the effective area E_{Eff} as the circumference of the pad multiplied by $R_{Eff}(z)$

$$A_{Eff}(z) \approx \pi D_{pad} \cdot R_{Eff}(z) = z \cdot \pi D_{pad} \tan\theta_{Max} \tag{7}$$

The effective area (green region in figure 6 right) is a function of the depth of the emission *z*. This implies that the emission is also a monotonic function in *z*, decreasing as the depth *z* decreases. As a result, emission closer to the contact is expected to be weaker than emission further away from the contact, as is observed in experiment.

Estimating reduction of $g^{(2)}(\tau=0,T)$ correlation peak due to one photon emission and losses and fitting

One-photon emission is assumed to behave as a Poisson distribution, with some one-photon rate registered at the detector Γ_{1P}. In order to register a false correlation event, two photons or more are required. The total probability is given by the following sum

$$\mathbb{P}(n \geq 2) = \sum_{n=2}^{\infty} \frac{e^{-\lambda}\lambda^n}{n!} \approx \frac{e^{-\lambda}\lambda^2}{2}, \lambda \leq 1 \quad (8)$$

Where $\lambda = \Gamma_{1P}\delta t$, with δt being a measurement time bin, given by the detector jitter of ~350 ps . In our experiment, $\Gamma_{1P} = 50 \times 10^3\ s^{-1}$, resulting in photon numbers higher than two being negligible, implying that $\mathbb{P}(n \geq 2) \approx \mathbb{P}(2)$. The contribution of the one-photon emission to the correlations can be given roughly as

$$N_{1P} = \frac{1}{2} \times \mathbb{P}(2) \times \frac{T_{Int}}{\Delta T_{dead}} \quad (9)$$

Where $T_{Int}, \Delta T_{dead}$ is the total integration time and the detector dead time (~20 ns). The factor of ½ is obtained from the beamsplitter, assuming 50:50 splitting. In practice, the one and two-photon rates arriving at the detectors suffer from losses along the way. Defining a transmission parameter $0 \leq t \leq 1$, we can write that $\Gamma_{1P} = t\Gamma_{1P-Emitted}$. For the two-photon emission, we assume a rate $\Gamma_{2P-Emitted}$. The contribution of the two-photon emission to the correlation is given by

$$N_{2P} = \frac{1}{2} \times t^2 \times \Gamma_{2P-Emitted} \times T_{Int} \quad (10)$$

Where the transmission is squared as both photons must arrive at the two detectors. The factor of ½ is obtained from the beamsplitter. Because the two photons are assumed to be

arriving together, the dead time of the detectors has no effect on the result. The correlation ratio can be obtained from the two rates

$$\frac{N_{2P}+N_{1P}}{N_{1P}} = 1+\frac{\frac{1}{2}\times t^2\times\Gamma\times T}{\frac{1}{2}\times\mathbb{P}(2)\times\frac{T}{\Delta T_{dead}}} = 1+\frac{2t^2\Gamma\Delta T_{dead}}{e^{-(t\Gamma\delta t)}(t\Gamma\delta t)^2}$$
$$= 1+e^{(t\Gamma\delta t)}\times\frac{\Delta T_{dead}}{\Gamma\delta t^2} \quad (11)$$

Where Γ is the detected rate for either one-photon or two-photon emission, which are equal based on the spectral features of the device being on the same order of magnitude. Based on the experimental results for the one-photon rates and the dead time of the detectors, we obtain an approximate transmission factor of

$$t = 2.6\times 10^{-3} \quad (12)$$

The loss factor is evaluated as $1-t=0.9974$ or ~99.7%. Prior to the application of loss and the addition of one-photon emission, the two-photon emission is perfectly correlated, implying a large 2nd order coherence peak, which equals the number of emitted two-photon pairs over the time span of the measurement. However, as a result of the loss factor and one-photon emission, the peak is drastically reduced, resulting in a value of 1.06 observed in our experiment.

While correlation peak value is small, it is still considerably above the noise floor of the measurements, as shown in the figure below. The two-photon correlations are predicted to scale like the two-photon emission rate, that is with $\Delta^2(T)$[13]. Thus, the calculated dependence is

$$g^{(2)}(\tau=0,T)=1+g_{Max}\cdot\Delta^{2}(T) \tag{13}$$

Where T_c,g_{Max} are the critical temperature and maximum correlation value at τ=0. The result obtained is T_c~6.08 K and g_{Max}~0.06, in good agreement with the experiment.

The measured $g^{(2)}(\tau,T)$ curves were normalized with respect to $g^{(2)}(\tau,T=10\ K)$ (Figure S7). For our measurements, because the photon count rates change with temperature, normalization is important in order to properly observe a change in $g^{(2)}(\tau,T)$. In addition, we present a clear trend of evolving correlations with temperature, which agrees well with the theoretical calculations.

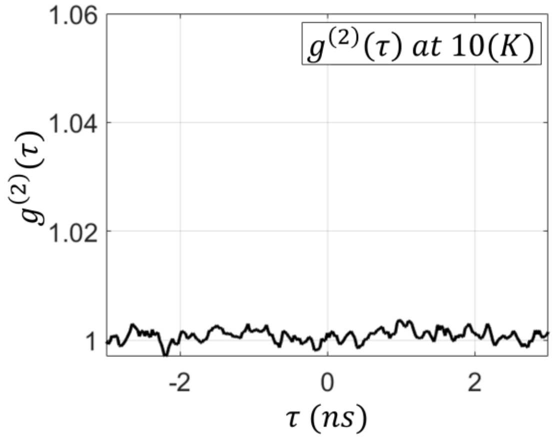

Figure S7 - ***Photon coincidence measurements of superlattice SLED emission at 10K:*** *The $g^2(\tau,T)$ curve was taken at 10 K and serves as the normalization curve for the reminder of the curves. The value of $g^2(\tau,T=3.6\ K)$ is ~1.04, much above the noise floor of the measurement.*

References

1 Blonder, G. E., Tinkham, M. & Klapwijk, T. M. Transition from metallic to tunneling regimes in superconducting microconstrictions: Excess current, charge imbalance, and supercurrent conversion. *Physical Review B* **25**, 4515-4532 (1982).

2 Bouscher, S., Winik, R. & Hayat, A. Andreev reflection enhancement in semiconductor-superconductor structures. *Physical Review B* **97**, 054512 (2018).

3 Bouscher, S. et al. Enhanced cooper-pair injection into a semiconductor structure by resonant tunneling. *Physical Review Letters* **128**, 127701 (2022).

4 Mattheiss, L. F. Electronic structure of niobium and tantalum. *Physical Review B* **1**, 373-380 (1970).

5 Levinshtein, M.S., Rumyantsev, S., Shur. M. Handbook series on semiconductor parameters Vol. 2. World Scientific (1997).

6 Karim, D. P., Ketterson, J. B. & Crabtree, G. W. A de Haas-van Alphen study of niobium: Fermi surface, cyclotron effective masses, and magnetic breakdown effects. *Journal of Low Temperature Physics* **30**, 389-423 (1978).

7 Büttiker, M. et al. Generalized many-channel conductance formula with application to small rings. *Physical Review B* **31**, 6207-6215 (1985).

8 Ihn, T. Semiconductor Nanostructures: Quantum States and Electronic Transport. (Oxford: Oxford University Press, 2009).

9 Kashiwaya, S. et al. Theory for tunneling spectroscopy of anisotropic superconductors. *Physical Review B* **53**, 2667-2676 (1996).

10 Kiphart, D. et al. Investigations of proximity-induced superconductivity in the topological insulator Bi_2Te_3 by microRaman spectroscopy. *Scientific Reports* **11**, 22980 (2021).

11 Yamashita, T. et al. Andreev reflection in ferromagnet/superconductor/ferromagnet double junction systems. *Physical Review B* **67**, 094515 (2003).

12 Flötotto, D. et al. Superconducting pairing of topological surface states in bismuth selenide films on niobium. *Science Advances* **4**, eaar7214 (2018).

13 Hayat, A. et al. Cooper-pair-based photon entanglement without isolated emitters. *Physical Review B*. **89**, 094508 (2014).
